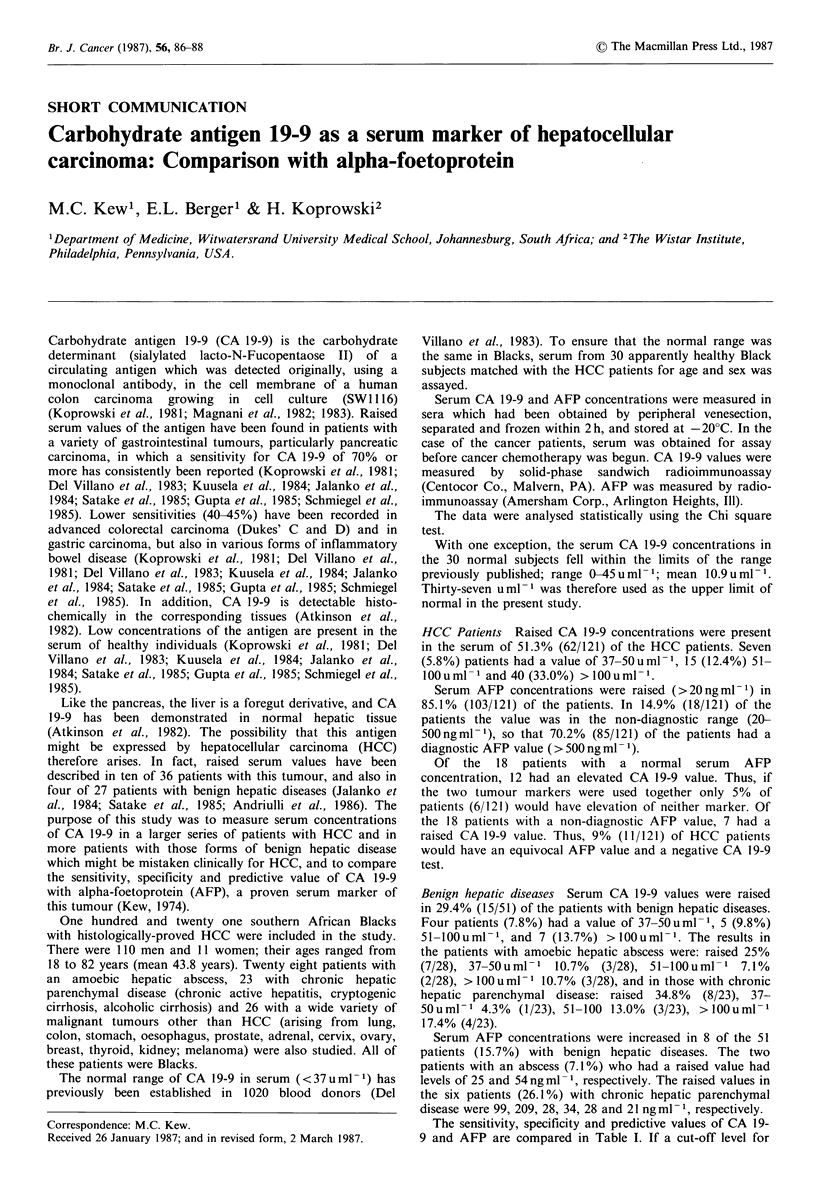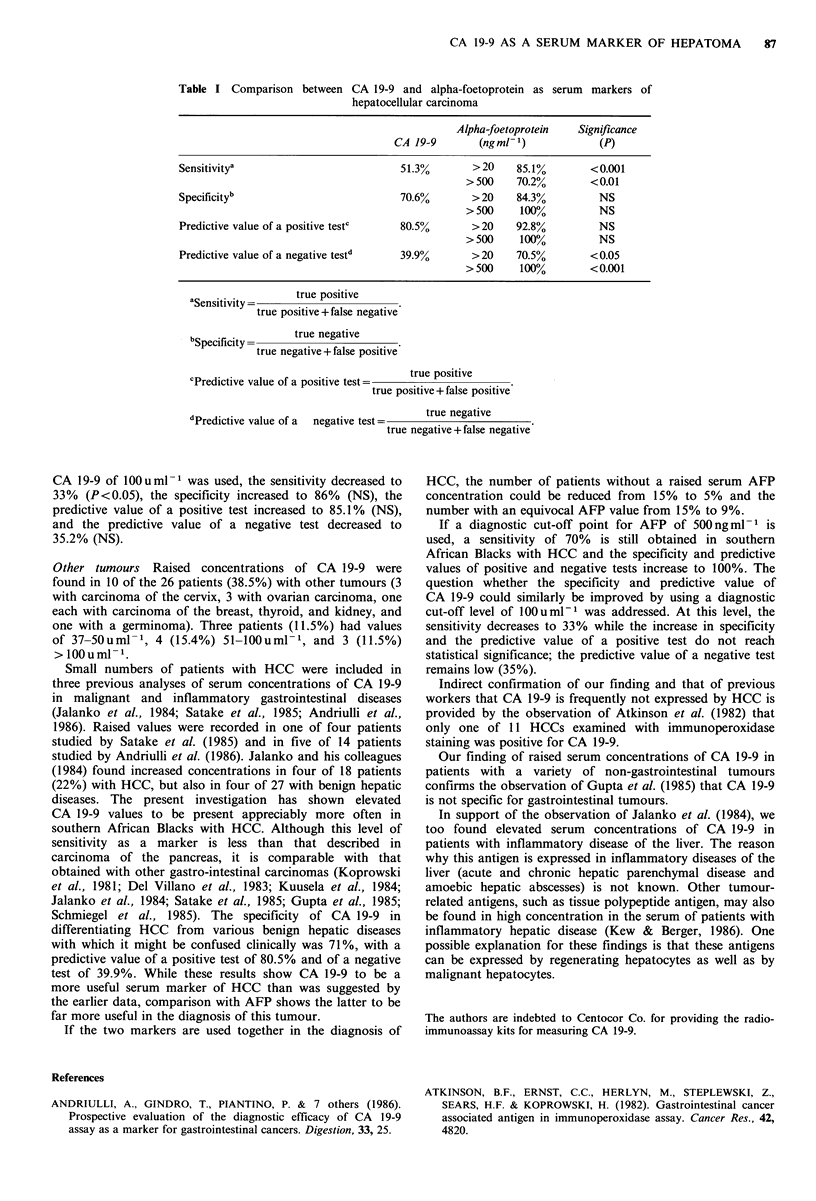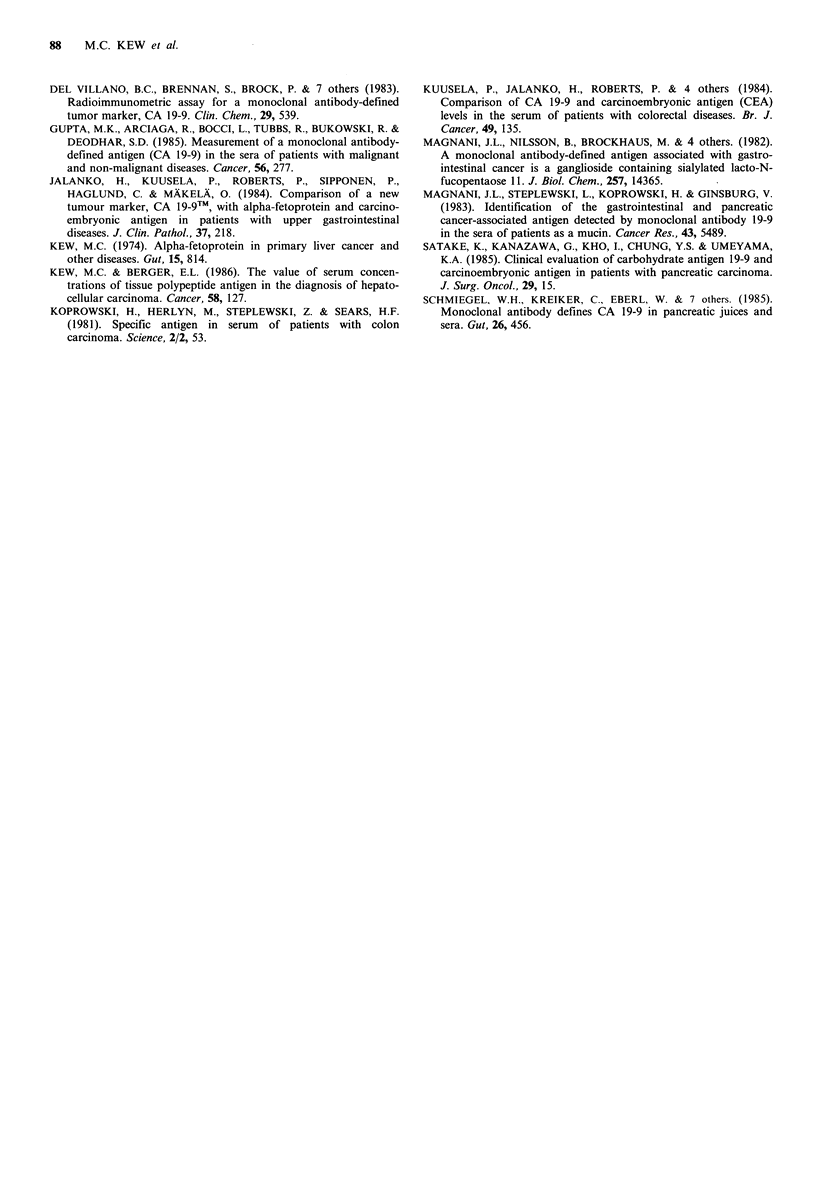# Carbohydrate antigen 19-9 as a serum marker of hepatocellular carcinoma: comparison with alpha-foetoprotein.

**DOI:** 10.1038/bjc.1987.160

**Published:** 1987-07

**Authors:** M. C. Kew, E. L. Berger, H. Koprowski


Br. J. Cancer (1987), 56, 86–88 © The Macmillan Press Ltd., 1987

SHORT COMMUNICATION

### Carbohydrate antigen 19-9 as a serum marker of hepatocellular carcinoma: Comparison with alpha-foetoprotein

M.C. Kew[1], E.L. Berger[1] & H. Koprowski[2]

[1]*Department of Medicine, Witwatersrand University Medical School, Johannesburg, South Africa; and* [2]*The Wistar Institute, Philadelphia, Pennsylvania, USA.*

Carbohydrate antigen 19-9 (CA 19-9) is the carbohydrate determinant (sialylated lacto-N-Fucopentaose II) of a circulating antigen which was detected originally, using a monoclonal antibody, in the cell membrane of a human colon carcinoma growing in cell culture (SW1116) (Koprowski *et al.*, 1981; Magnani *et al.*, 1982; 1983). Raised serum values of the antigen have been found in patients with a variety of gastrointestinal tumours, particularly pancreatic carcinoma, in which a sensitivity for CA 19-9 of 70% or more has consistently been reported (Koprowski *et al.*, 1981; Del Villano *et al.*, 1983; Kuusela *et al.*, 1984; Jalanko *et al.*, 1984; Satake *et al.*, 1985; Gupta *et al.*, 1985; Schmiegel *et al.*, 1985). Lower sensitivities (40–45%) have been recorded in advanced colorectal carcinoma (Dukes' C and D) and in gastric carcinoma, but also in various forms of inflammatory bowel disease (Koprowski *et al.*, 1981; Del Villano *et al.*, 1981; Del Villano *et al.*, 1983; Kuusela *et al.*, 1984; Jalanko *et al.*, 1984; Satake *et al.*, 1985; Gupta *et al.*, 1985; Schmiegel *et al.*, 1985). In addition, CA 19-9 is detectable histochemically in the corresponding tissues (Atkinson *et al.*, 1982). Low concentrations of the antigen are present in the serum of healthy individuals (Koprowski *et al.*, 1981; Del Villano *et al.*, 1983; Kuusela *et al.*, 1984; Jalanko *et al.*, 1984; Satake *et al.*, 1985; Gupta *et al.*, 1985; Schmiegel *et al.*, 1985).

Like the pancreas, the liver is a foregut derivative, and CA 19-9 has been demonstrated in normal hepatic tissue (Atkinson *et al.*, 1982). The possibility that this antigen might be expressed by hepatocellular carcinoma (HCC) therefore arises. In fact, raised serum values have been described in ten of 36 patients with this tumour, and also in four of 27 patients with benign hepatic diseases (Jalanko *et al.*, 1984; Satake *et al.*, 1985; Andriulli *et al.*, 1986). The purpose of this study was to measure serum concentrations of CA 19-9 in a larger series of patients with HCC and in more patients with those forms of benign hepatic disease which might be mistaken clinically for HCC, and to compare the sensitivity, specificity and predictive value of CA 19-9 with alpha-foetoprotein (AFP), a proven serum marker of this tumour (Kew, 1974).

One hundred and twenty one southern African Blacks with histologically-proved HCC were included in the study. There were 110 men and 11 women; their ages ranged from 18 to 82 years (mean 43.8 years). Twenty eight patients with an amoebic hepatic abscess, 23 with chronic hepatic parenchymal disease (chronic active hepatitis, cryptogenic cirrhosis, alcoholic cirrhosis) and 26 with a wide variety of malignant tumours other than HCC (arising from lung, colon, stomach, oesophagus, prostate, adrenal, cervix, ovary, breast, thyroid, kidney; melanoma) were also studied. All of these patients were Blacks.

The normal range of CA 19-9 in serum ($<37\,u\,ml^{-1}$) has previously been established in 1020 blood donors (Del Villano *et al.*, 1983). To ensure that the normal range was the same in Blacks, serum from 30 apparently healthy Black subjects matched with the HCC patients for age and sex was assayed.

Serum CA 19-9 and AFP concentrations were measured in sera which had been obtained by peripheral venesection, separated and frozen within 2 h, and stored at −20°C. In the case of the cancer patients, serum was obtained for assay before cancer chemotherapy was begun. CA 19-9 values were measured by solid-phase sandwich radioimmunoassay (Centocor Co., Malvern, PA). AFP was measured by radioimmunoassay (Amersham Corp., Arlington Heights, Ill).

The data were analysed statistically using the Chi square test.

With one exception, the serum CA 19-9 concentrations in the 30 normal subjects fell within the limits of the range previously published; range $0–45\,u\,ml^{-1}$; mean $10.9\,u\,ml^{-1}$. Thirty-seven $u\,ml^{-1}$ was therefore used as the upper limit of normal in the present study.

*HCC Patients* Raised CA 19-9 concentrations were present in the serum of 51.3% (62/121) of the HCC patients. Seven (5.8%) patients had a value of $37–50\,u\,ml^{-1}$, 15 (12.4%) $51–100\,u\,ml^{-1}$ and 40 (33.0%) $>100\,u\,ml^{-1}$.

Serum AFP concentrations were raised ($>20\,ng\,ml^{-1}$) in 85.1% (103/121) of the patients. In 14.9% (18/121) of the patients the value was in the non-diagnostic range ($20–500\,ng\,ml^{-1}$), so that 70.2% (85/121) of the patients had a diagnostic AFP value ($>500\,ng\,ml^{-1}$).

Of the 18 patients with a normal serum AFP concentration, 12 had an elevated CA 19-9 value. Thus, if the two tumour markers were used together only 5% of patients (6/121) would have elevation of neither marker. Of the 18 patients with a non-diagnostic AFP value, 7 had a raised CA 19-9 value. Thus, 9% (11/121) of HCC patients would have an equivocal AFP value and a negative CA 19-9 test.

*Benign hepatic diseases* Serum CA 19-9 values were raised in 29.4% (15/51) of the patients with benign hepatic diseases. Four patients (7.8%) had a value of $37–50\,u\,ml^{-1}$, 5 (9.8%) $51–100\,u\,ml^{-1}$, and 7 (13.7%) $>100\,u\,ml^{-1}$. The results in the patients with amoebic hepatic abscess were: raised 25% (7/28), $37–50\,u\,ml^{-1}$ 10.7% (3/28), $51–100\,u\,ml^{-1}$ 7.1% (2/28), $>100\,u\,ml^{-1}$ 10.7% (3/28), and in those with chronic hepatic parenchymal disease: raised 34.8% (8/23), $37–50\,u\,ml^{-1}$ 4.3% (1/23), 51–100 13.0% (3/23), $>100\,u\,ml^{-1}$ 17.4% (4/23).

Serum AFP concentrations were increased in 8 of the 51 patients (15.7%) with benign hepatic diseases. The two patients with an abscess (7.1%) who had a raised value had levels of 25 and $54\,ng\,ml^{-1}$, respectively. The raised values in the six patients (26.1%) with chronic hepatic parenchymal disease were 99, 209, 28, 34, 28 and $21\,ng\,ml^{-1}$, respectively.

The sensitivity, specificity and predictive values of CA 19-9 and AFP are compared in Table I. If a cut-off level for

Correspondence: M.C. Kew.
Received 26 January 1987; and in revised form, 2 March 1987.

**Table I** Comparison between CA 19-9 and alpha-foetoprotein as serum markers of hepatocellular carcinoma

| | *CA 19-9* | *Alpha-foetoprotein ($ng\,ml^{-1}$)* | | *Significance (P)* |
|---|---|---|---|---|
| Sensitivity[a] | 51.3% | >20 | 85.1% | <0.001 |
| | | >500 | 70.2% | <0.01 |
| Specificity[b] | 70.6% | >20 | 84.3% | NS |
| | | >500 | 100% | NS |
| Predictive value of a positive test[c] | 80.5% | >20 | 92.8% | NS |
| | | >500 | 100% | NS |
| Predictive value of a negative test[d] | 39.9% | >20 | 70.5% | <0.05 |
| | | >500 | 100% | <0.001 |

[a]Sensitivity $= \frac{\text{true positive}}{\text{true positive} + \text{false negative}}$.

[b]Specificity $= \frac{\text{true negative}}{\text{true negative} + \text{false positive}}$.

[c]Predictive value of a positive test $= \frac{\text{true positive}}{\text{true positive} + \text{false positive}}$.

[d]Predictive value of a negative test $= \frac{\text{true negative}}{\text{true negative} + \text{false negative}}$.

CA 19-9 of $100\,u\,ml^{-1}$ was used, the sensitivity decreased to 33% ($P<0.05$), the specificity increased to 86% (NS), the predictive value of a positive test increased to 85.1% (NS), and the predictive value of a negative test decreased to 35.2% (NS).

*Other tumours* Raised concentrations of CA 19-9 were found in 10 of the 26 patients (38.5%) with other tumours (3 with carcinoma of the cervix, 3 with ovarian carcinoma, one each with carcinoma of the breast, thyroid, and kidney, and one with a germinoma). Three patients (11.5%) had values of $37\text{–}50\,u\,ml^{-1}$, 4 (15.4%) $51\text{–}100\,u\,ml^{-1}$, and 3 (11.5%) $>100\,u\,ml^{-1}$.

Small numbers of patients with HCC were included in three previous analyses of serum concentrations of CA 19-9 in malignant and inflammatory gastrointestinal diseases (Jalanko *et al.*, 1984; Satake *et al.*, 1985; Andriulli *et al.*, 1986). Raised values were recorded in one of four patients studied by Satake *et al.* (1985) and in five of 14 patients studied by Andriulli *et al.* (1986). Jalanko and his colleagues (1984) found increased concentrations in four of 18 patients (22%) with HCC, but also in four of 27 with benign hepatic diseases. The present investigation has shown elevated CA 19-9 values to be present appreciably more often in southern African Blacks with HCC. Although this level of sensitivity as a marker is less than that described in carcinoma of the pancreas, it is comparable with that obtained with other gastro-intestinal carcinomas (Koprowski *et al.*, 1981; Del Villano *et al.*, 1983; Kuusela *et al.*, 1984; Jalanko *et al.*, 1984; Satake *et al.*, 1985; Gupta *et al.*, 1985; Schmiegel *et al.*, 1985). The specificity of CA 19-9 in differentiating HCC from various benign hepatic diseases with which it might be confused clinically was 71%, with a predictive value of a positive test of 80.5% and of a negative test of 39.9%. While these results show CA 19-9 to be a more useful serum marker of HCC than was suggested by the earlier data, comparison with AFP shows the latter to be far more useful in the diagnosis of this tumour.

If the two markers are used together in the diagnosis of HCC, the number of patients without a raised serum AFP concentration could be reduced from 15% to 5% and the number with an equivocal AFP value from 15% to 9%.

If a diagnostic cut-off point for AFP of $500\,ng\,ml^{-1}$ is used, a sensitivity of 70% is still obtained in southern African Blacks with HCC and the specificity and predictive values of positive and negative tests increase to 100%. The question whether the specificity and predictive value of CA 19-9 could similarly be improved by using a diagnostic cut-off level of $100\,u\,ml^{-1}$ was addressed. At this level, the sensitivity decreases to 33% while the increase in specificity and the predictive value of a positive test do not reach statistical significance; the predictive value of a negative test remains low (35%).

Indirect confirmation of our finding and that of previous workers that CA 19-9 is frequently not expressed by HCC is provided by the observation of Atkinson *et al.* (1982) that only one of 11 HCCs examined with immunoperoxidase staining was positive for CA 19-9.

Our finding of raised serum concentrations of CA 19-9 in patients with a variety of non-gastrointestinal tumours confirms the observation of Gupta *et al.* (1985) that CA 19-9 is not specific for gastrointestinal tumours.

In support of the observation of Jalanko *et al.* (1984), we too found elevated serum concentrations of CA 19-9 in patients with inflammatory disease of the liver. The reason why this antigen is expressed in inflammatory diseases of the liver (acute and chronic hepatic parenchymal disease and amoebic hepatic abscesses) is not known. Other tumour-related antigens, such as tissue polypeptide antigen, may also be found in high concentration in the serum of patients with inflammatory hepatic disease (Kew & Berger, 1986). One possible explanation for these findings is that these antigens can be expressed by regenerating hepatocytes as well as by malignant hepatocytes.

The authors are indebted to Centocor Co. for providing the radioimmunoassay kits for measuring CA 19-9.

### References

ANDRIULLI, A., GINDRO, T., PIANTINO, P. & 7 others (1986). Prospective evaluation of the diagnostic efficacy of CA 19-9 assay as a marker for gastrointestinal cancers. *Digestion*, **33**, 25.

ATKINSON, B.F., ERNST, C.C., HERLYN, M., STEPLEWSKI, Z., SEARS, H.F. & KOPROWSKI, H. (1982). Gastrointestinal cancer associated antigen in immunoperoxidase assay. *Cancer Res.*, **42**, 4820.

DEL VILLANO, B.C., BRENNAN, S., BROCK, P. & 7 others (1983). Radioimmunometric assay for a monoclonal antibody-defined tumor marker, CA 19-9. *Clin. Chem.*, **29**, 539.

GUPTA, M.K., ARCIAGA, R., BOCCI, L., TUBBS, R., BUKOWSKI, R. & DEODHAR, S.D. (1985). Measurement of a monoclonal antibody-defined antigen (CA 19-9) in the sera of patients with malignant and non-malignant diseases. *Cancer*, **56**, 277.

JALANKO, H., KUUSELA, P., ROBERTS, P., SIPPONEN, P., HAGLUND, C. & MÄKELÄ, O. (1984). Comparison of a new tumour marker, CA 19-9™, with alpha-fetoprotein and carcinoembryonic antigen in patients with upper gastrointestinal diseases. *J. Clin. Pathol.*, **37**, 218.

KEW, M.C. (1974). Alpha-fetoprotein in primary liver cancer and other diseases. *Gut*, **15**, 814.

KEW, M.C. & BERGER, E.L. (1986). The value of serum concentrations of tissue polypeptide antigen in the diagnosis of hepatocellular carcinoma. *Cancer*, **58**, 127.

KOPROWSKI, H., HERLYN, M., STEPLEWSKI, Z. & SEARS, H.F. (1981). Specific antigen in serum of patients with colon carcinoma. *Science*, **2/2**, 53.